# Supplementary figures and images for: A random-sampling approach to track cell divisions in time-lapse fluorescence microscopy
Source: Plant Methods. 2021 Mar 8;17:25. doi: 10.1186/s13007-021-00723-8 (PMC7941913; doi:10.1186/s13007-021-00723-8)

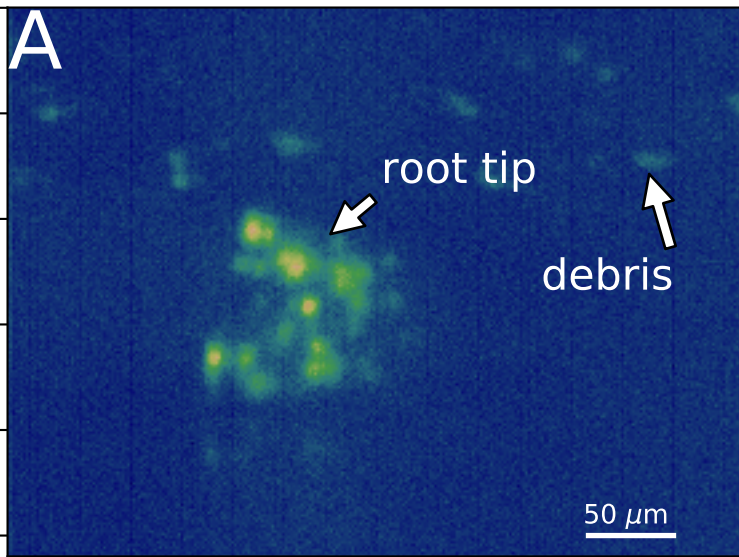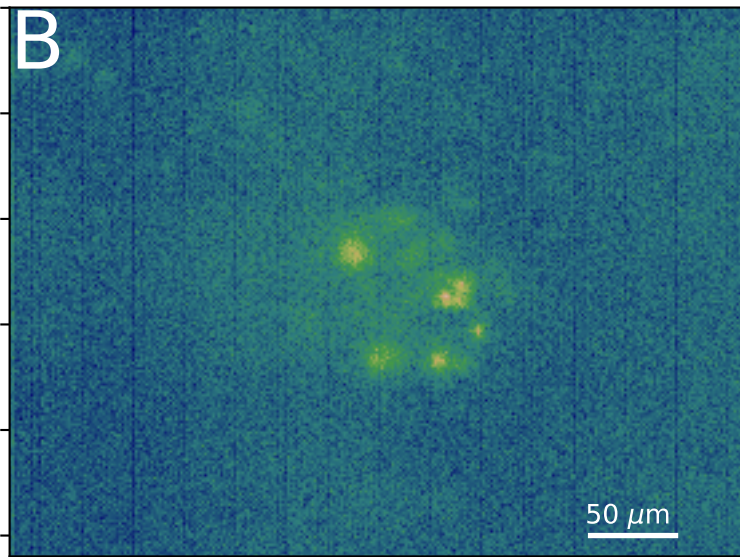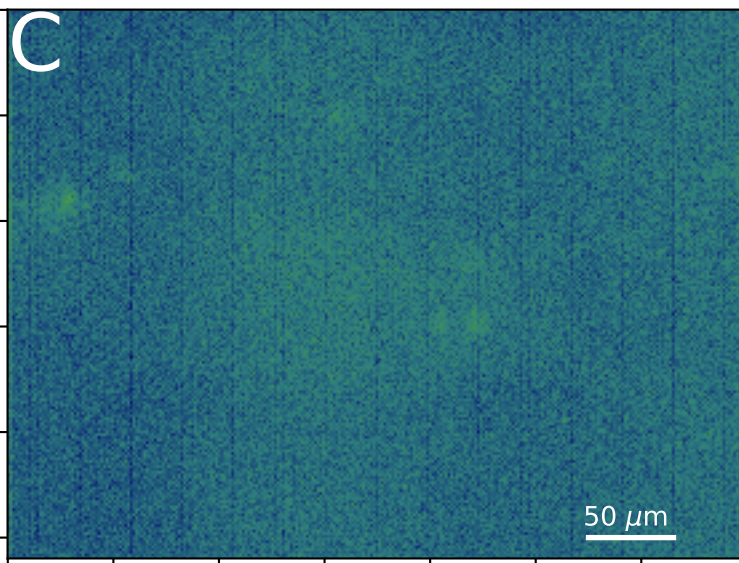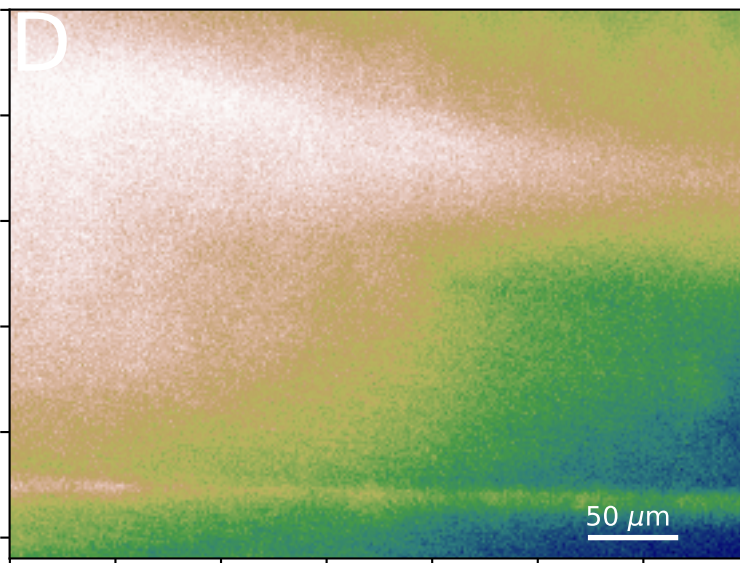

Supplement: Supplementary file 1 — Additional file 1: Fig S1. Four representative frames: A, “good” image; B, noisy image; C, noisy image with a low number of objects and some noise; D, over-saturated image to be marked as degenerate. [file 13007_2021_723_MOESM1_ESM.pdf]

Average tracking score for sample roots

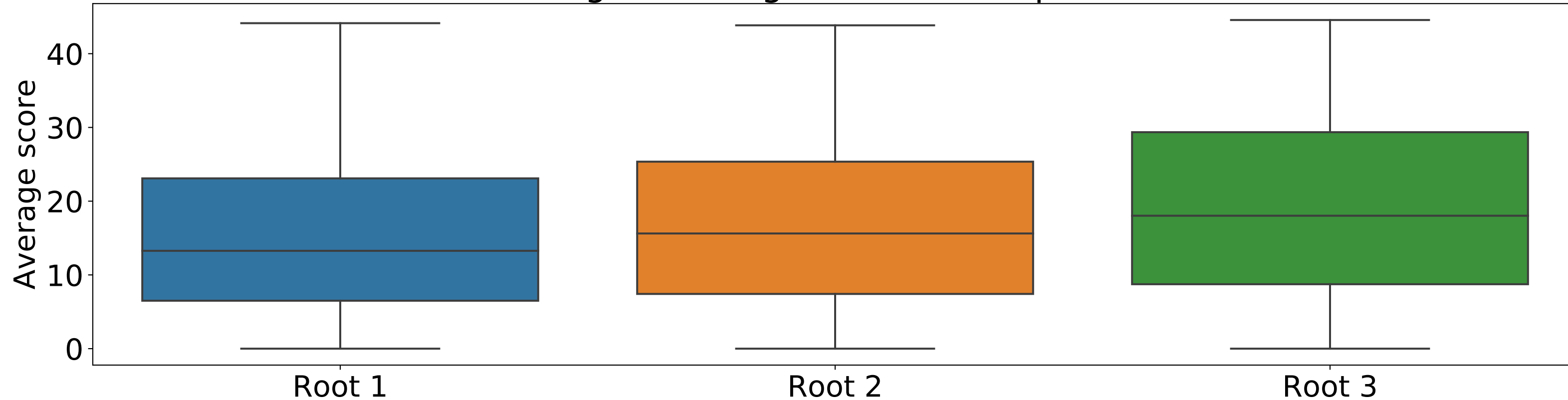

Supplement: Supplementary file 2 — Additional file 3: Fig S3. Performance for multiple root samples: box plots show the meantracker score, averaged over 200 frames for each root. [file 13007_2021_723_MOESM2_ESM.pdf]

Comparing average detection rate for epsilon values

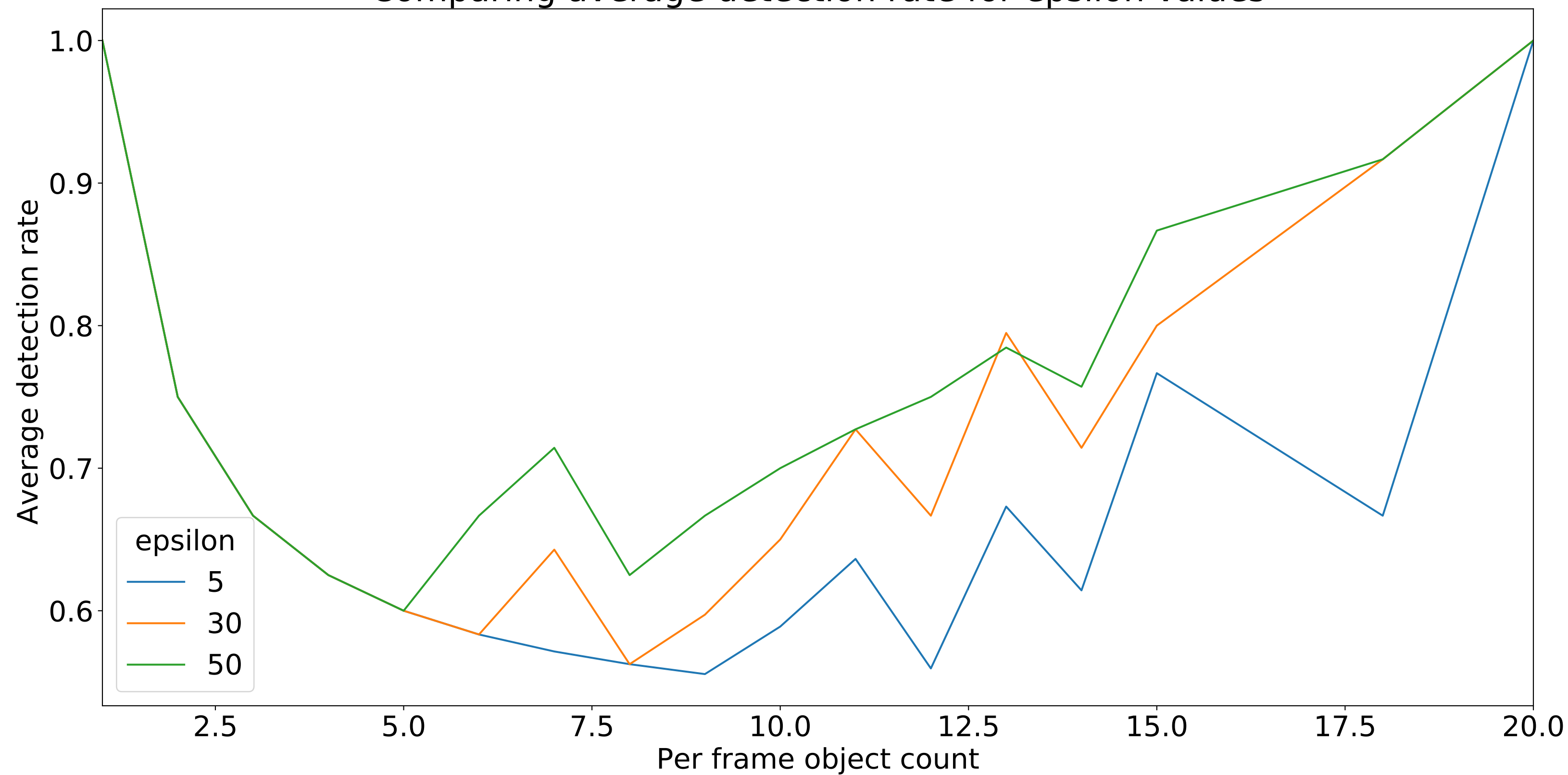

Supplement: Supplementary file 3 — Additional file 4: Fig S4. Average detection rate for 200 frames in each of the three sampleroots. [file 13007_2021_723_MOESM3_ESM.pdf]

Comparing detection rate standard deviation for epsilon values

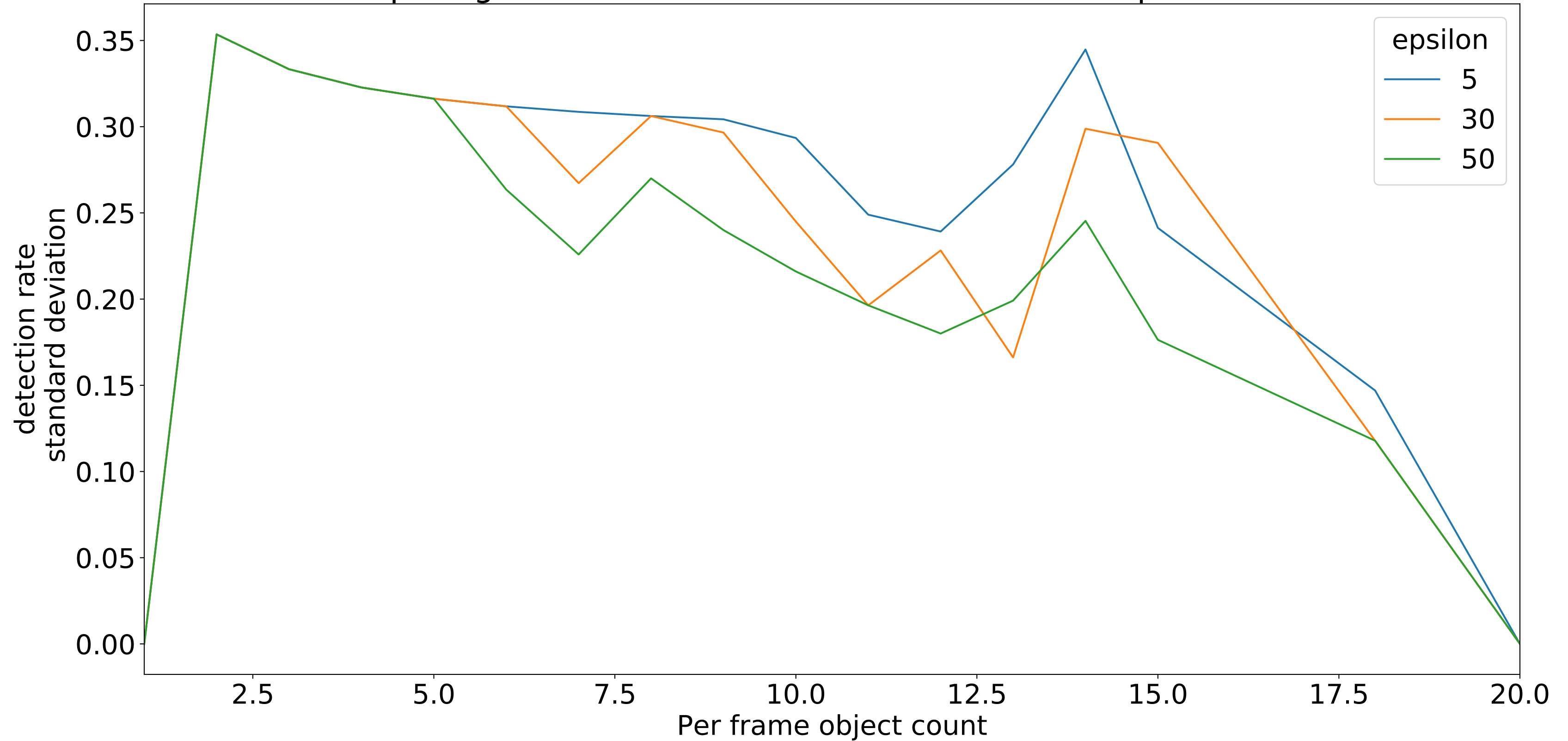

Supplement: Supplementary file 4 — Additional file 5: Fig S5. Standard deviation of detection rate for 200 frames in each of thethree sample roots. [file 13007_2021_723_MOESM4_ESM.pdf]

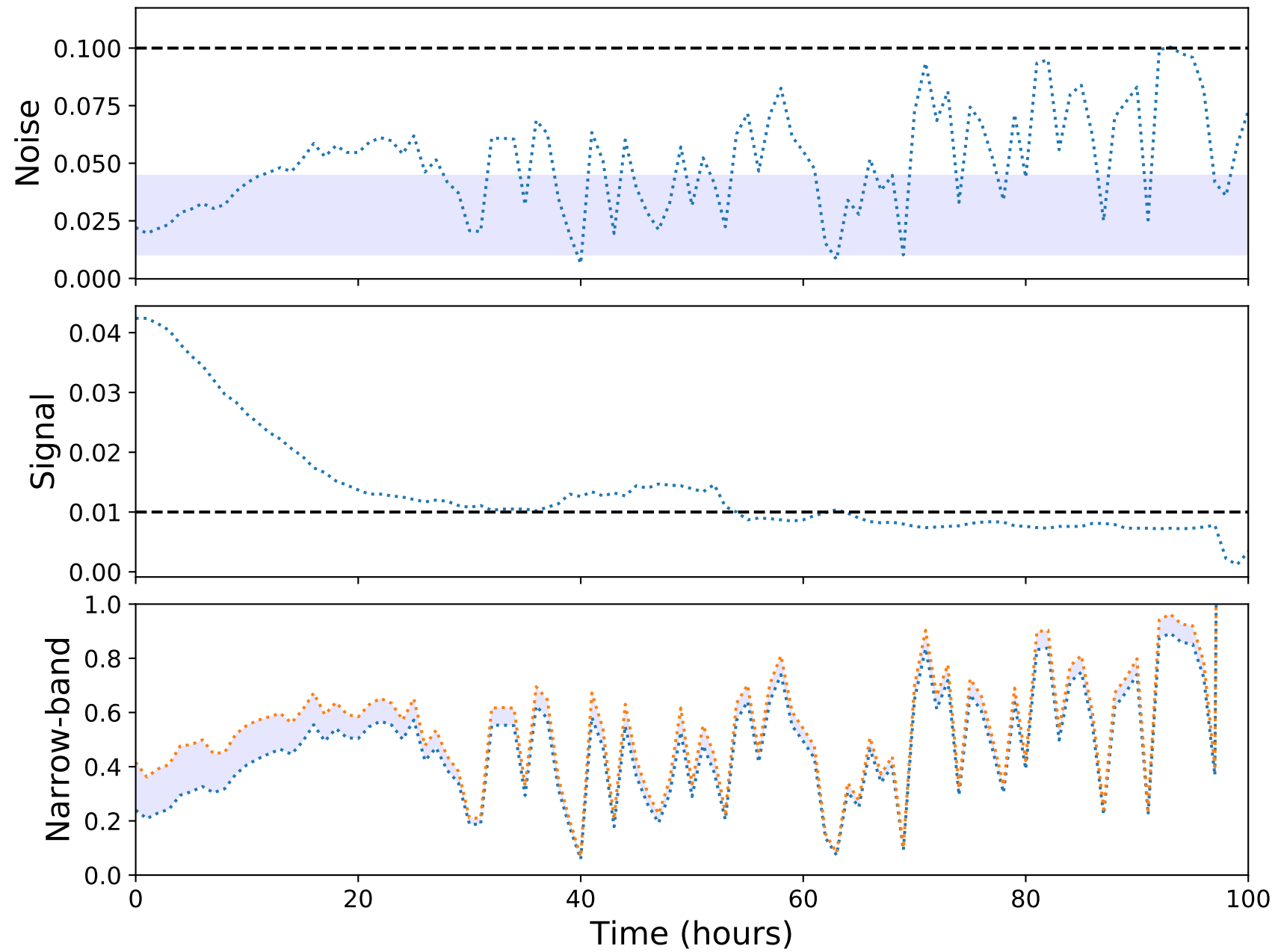

Supplement: Supplementary file 5 — Additional file 2: Fig S2. Variation in frame properties: a quantitative analysis over 100 consecutive sample frames from one representative root. The Signal and Noise levels are plotted with threshold values. The Narrow-band plot shows the region between the 95th and 99th percentile ranges of the image histogram. Notably, these values fluctuate dramatically. [file 13007_2021_723_MOESM5_ESM.pdf]

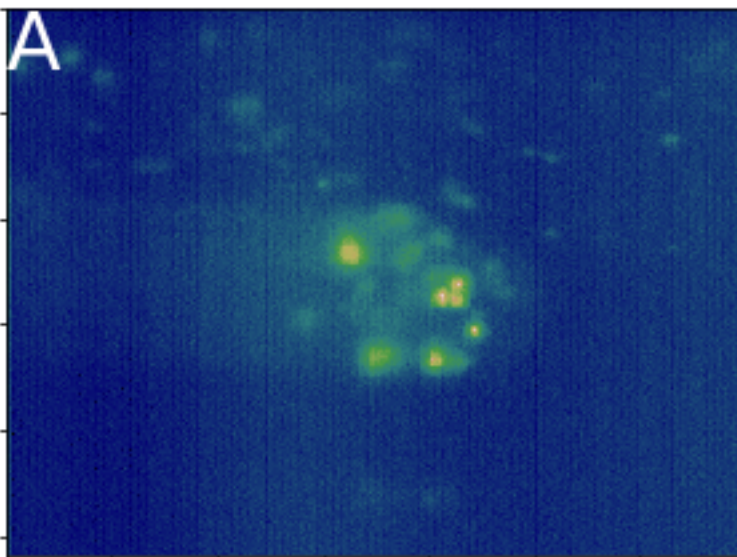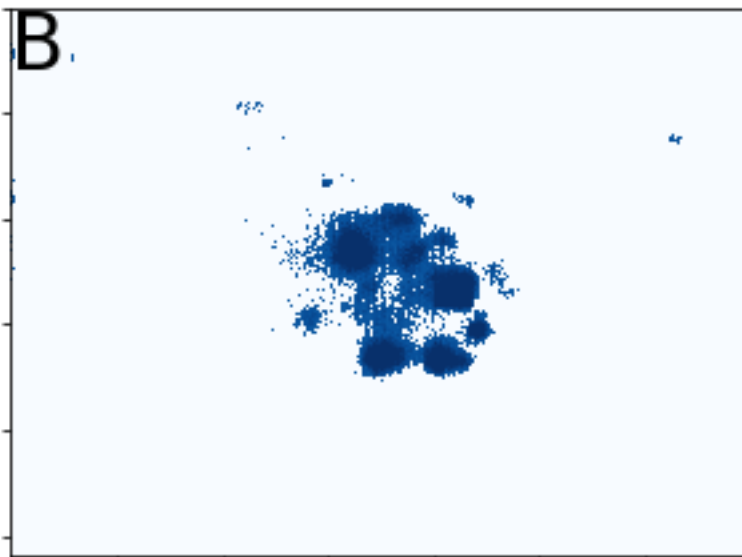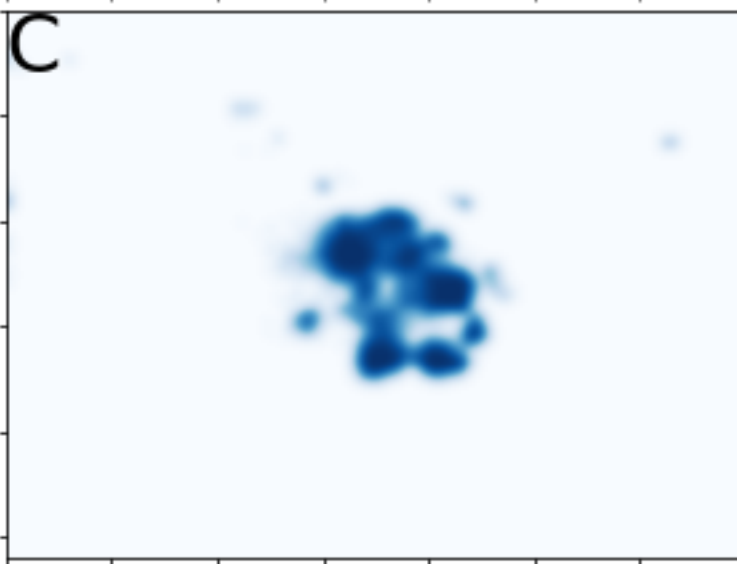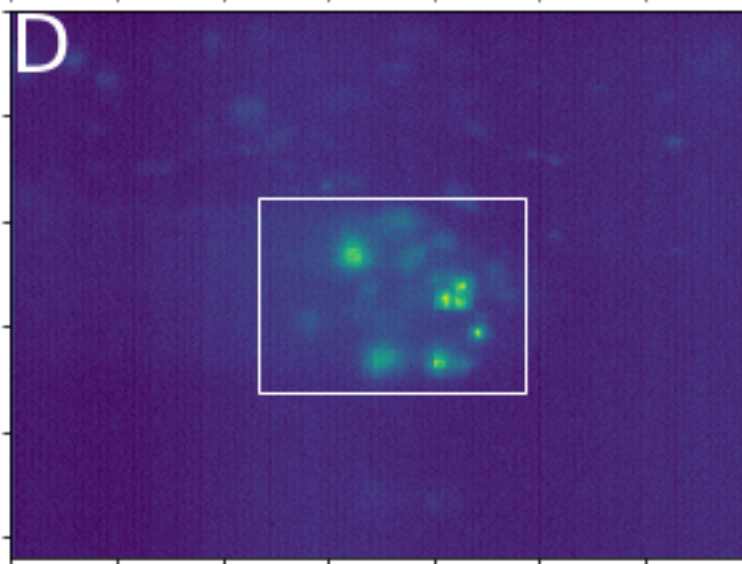

Supplement: Supplementary file 6 — Additional file 6: Fig S6. Isolating the Region of Interest (ROI): a narrow-band filter (B) and aggressive Gaussian smoothing (C) is used to find the largest connected com-ponent in the narrow band of the data corresponding to the region of activity in the root tip (D). [file 13007_2021_723_MOESM6_ESM.pdf]
